# Binocular Vision Anomalies: An Update on Prevalence, Impact, Technological and Treatment Developments and Current Challenges

**DOI:** 10.1007/s44402-026-00119-y

**Published:** 2026-06-15

**Authors:** Riccardo Cheloni, Brendan T. Barrett

**Affiliations:** 1 School of Pharmacy, Optometry & Medical Sciences, University of Bradford, Bradford, UK; 2 Moorfields Eye Hospital NHS Foundation Trust, London, UK

**Keywords:** Amblyopia, Non-strabismic binocular vision anomalies, Strabismus, Vergence disorders

## Abstract

**Purpose:**

To identify key recent research in the field of binocular vision anomalies, highlight important advances and identify where new developments are emerging. This allows identification of gaps in our knowledge, which may inform future research efforts.

**Methods:**

A narrative review of the peer-reviewed literature on amblyopia, strabismus and non-strabismic binocular vision anomalies (NSBVA) was carried out under the following headings: prevalence, impact and new developments (e.g., in technology, treatment and disease understanding).

**Results:**

The prevalence of amblyopia and strabismus continues to be studied intensively in different geographical regions and amongst different ethnicities, with results revealing large variations in rates of esotropia versus exotropia. Studies of NSBVA prevalence frequently suffer from major methodological limitations, leaving uncertainty about their prevalence and impact. This stems from variability in diagnostic criteria and an uncertain link between the conditions and the symptoms generated. There has been an upsurge in evaluations of the impact of strabismus and amblyopia. Along with their effect on vision, changes are seen in visuomotor and other functional task performance (e.g., reading). Additionally, there are important psychosocial impacts (e.g., in self-confidence), particularly when strabismus is present. Digital applications are emerging for both detecting and treating binocular anomalies, although much remains to be done to establish their acceptability to patients and overall usefulness. The quality of the research evidence guiding amblyopia treatment is much stronger than for strabismus, where evidence for the treatment of the condition is still lacking.

**Conclusions:**

With greater awareness of the need for robust research evidence, the knowledge gaps concerning binocular vision anomalies are becoming clearer. As high-quality research evidence emerges and armed with a greater understanding of their prevalence and impact, it is hoped that future management of these visual anomalies will be based more upon robust research evidence and less upon clinical consensus.

Key points

- The prevalence and impact of amblyopia and strabismus continue to be studied intensively and are now well understood. This contrasts with non-strabismic binocular vision anomalies, for which considerable uncertainty exists regarding their prevalence and impact.
- The quality of the research evidence guiding amblyopia treatment is much stronger than for strabismus and for non-strabismic binocular vision anomalies.
- There is growing awareness of the areas where research evidence in binocular vision research is lacking. As high-quality research evidence emerges, this should form the underlying basis for understanding and managing these conditions, rather than clinical consensus.

## Introduction

Binocular vision is a key attribute of the human visual system. Typically, reduced binocularity is associated with one or more developmental disorders of vision (e.g., amblyopia or strabismus), but it can also present as an acquired condition, with or without strabismus. Binocular disorders exist in many forms, and research is ongoing to understand their prevalence and the impact they exert on vision and visuomotor functions, as well as on psychosocial aspects. The efficacy with which binocular disorders can be treated is the subject of considerable research interest, particularly for certain conditions (e.g., amblyopia) and new advances are also being made in relation to detecting their presence and in understanding their underlying basis.

The purpose of this review is to provide updates on recent, clinically oriented research on the topic of binocular vision. The recent literature (2015 to the present time) was considered under the headings of the prevalence of different binocular conditions; the visual and non-visual impacts of living with them and new developments in relation to disease detection, understanding and treatment efficacy. In this review of these topics, amblyopia and strabismus were considered together and separately from the recent literature on non-strabismic binocular vision anomalies (NSBVA). The review concludes by highlighting what are considered to be the most promising areas of development and the areas where gaps continue to exist in our knowledge. Identifying these gaps may help to point to where future research efforts may usefully be directed.

## Strabismus and amblyopia

### Prevalence of strabismus

The overall prevalence of manifest strabismus is estimated to be between 0.8% and 6.8% [1], though many studies have indicated a prevalence of 2–3% [2–4]. Strabismus prevalence varies considerably between countries and ethnicities [2], and it may be that the overall prevalence has fallen in some parts of the world over the last three decades (e.g. [2, 5, 6]).

The prevalence of strabismus is critically dependent upon the population studied. For example, Perna et al. found meta-analytic evidence that the prevalence of strabismus is between four and five times higher in people with autism spectrum disorders compared with those without these disorders [7]. A high prevalence of strabismus exists in children and adolescents with cardiac health disease [8] and in people with cerebral palsy [9, 10], with Herron et al. reporting a prevalence of strabismus in those with cerebral palsy of 49.8% in high-income countries and 39.8% in lower-income countries [10]. Differences in prevalence between countries with different socio-economic standings may be related to eyecare provision or uptake. Interestingly, the distribution of esotropia and exotropia in patients with cerebral palsy largely reflects the horizontal strabismus type predominant in that particular ethnicity.

In Down syndrome, the prevalence of strabismus is around 30% [3, 11]. In populations which normally have more esotropia than exotropia (e.g., Caucasians), individuals with Down syndrome have a further increased bias towards esotropia. Similarly, in populations which normally exhibit more exotropia (e.g., West Africans, Asians and Hispanics), subjects with Down syndrome have a significantly lower ratio of esotropia to exotropia, compared with Caucasians with Down syndrome. It is important to stress that ethnic differences in the esotropia/exotropia ratio both in a population at large and clinical in sub-populations need to be considered against the refractive error characteristics of the population being studied, particularly in relation to the strong association between hyperopia and esotropia [12, 13]. However, an intriguing finding from a recent meta-analysis is that racial differences in interpupillary distance may also be associated with horizontal strabismus patterns, possibly even contributing to differences in the esotropia/exotropia ratio in different ethnicities [14].

Recent systematic reviews of prevalence data suggest that while the prevalence of strabismus in the general population is similar in males and females, strabismus prevalence amongst females is higher in clinic-based studies [2]. The extent of this female gender bias differs between geographic regions, and it is possible that it reflects a greater proportion of females with strabismus seeking health care or being brought to clinics compared to males with strabismus.

### Prevalence of amblyopia

Amblyopia is a leading cause of vision impairment, especially among children and young adults [15–17]. Most estimates examining the prevalence of amblyopia are between 2% and 5%, with differences between prevalence estimates being due mainly to the population studied and the definition of amblyopia being applied [18]. Whereas relatively recent large-scale reviews quote a prevalence range of 1.6–3.6% [19] or 1–3% [20], more recent systematic reviews suggest a somewhat lower prevalence, for example, 1.44% [16] and 1.36% [21].

With some exceptions [21], most investigations found no significant difference in the prevalence between genders [16]. The results from many studies suggest that differences may exist in prevalence between different geographical locations/ethnicities [15, 16, 22, 23], but this is not a universal finding [21].

Given that amblyopia is a treatable condition, in countries and regions where amblyopia is screened for and treated when found, the prevalence is seen to fall across the range of ages over which treatment is undertaken, whereas prevalence does not change with age when attempts to treat it have ceased [20]. This is consistent with the view that amblyopia prevalence is higher amongst socially disadvantaged and medically underserved communities [19].

### The significance and impact of strabismus and amblyopia

Owing to their frequent co-occurrence [15], separating the impact of strabismus and amblyopia is challenging. This section briefly reviews the impact of these conditions when they present both together and separately, with an emphasis on what recent research has revealed.

#### Binocular vision

Alongside the reduction in visual acuity (VA) that defines amblyopia, reduced binocularity as measured clinically is characteristic of strabismus, either with or without amblyopia [13, 24]. Recent work adds weight to two long-established but relatively under-researched topics, namely that a form of binocularity, albeit a degraded type, exists in individuals with amblyopia and/or strabismus [25–28], and that subtle deficits may exist in the fellow eye of an individual with amblyopia or in the fixating eye of someone with strabismus [29, 30].

Haraguchi et al. found that binocular and fellow eye VA were significantly worse in persons with amblyopia, whereas binocular and dominant eye VA in individuals with strabismus but without amblyopia did not differ from control subjects [31]. Supporting a view that a form of binocularity exists in these individuals, both amblyopic and strabismic subjects exhibited binocular summation (i.e., binocular VA was superior to fellow/dominant eye VA). This accords with earlier findings that both the magnitude of residual binocularity and interocular suppression may be linked with the monocular VA, which itself may provide clues to the aetiology of the vision loss [32]. Binocular summation has also been observed in other tasks in amblyopes, indicating that the non-dominant eye may contribute usefully when both eyes are open [28].

Regarding fellow-eye deficits, Birch et al. [29] found poorer performance on a motion-defined form task in the fellow eyes of 23% of children with residual amblyopia and in 20% of non-amblyopic children with treated strabismus or anisometropia. Motion-defined form thresholds reflect the ability to identify a two-dimensional shape defined by motion rather than luminance contrast. Of note is that thresholds were correlated with stereoacuity and, intriguingly, in the amblyopes, with the age when the child was first patched [29].

#### Reading and academic ability

Webber identified reduced reading ability amongst the deficits associated with amblyopia [33]. The results of Kelly et al. revealed slower reading in children with anisometropic amblyopia, and the magnitude of the deficits was associated with fixation instability and increased frequency of saccades [34]. Recent results support the view that reading rate is slower in both those with strabismus only and in individuals with both strabismus and amblyopia [35]. Also, slower reading performance in the latter study was associated with poorer fine motor skills [35]. Ridha et al. reported that strabismus surgery may improve the reading ability of school-age children [36].

Kelly et al. [37] found that amblyopic children and children with non-amblyopic strabismus took ~28% longer to complete a task that involved transferring the correct answers from a standardised reading achievement test booklet to a multiple-choice answer form. Interestingly, completion times were not associated with aetiology, VA or stereoacuity.

Yang et al. found that, alongside reduced binocular function and visual information processing skills, reading ability is reduced in children with intermittent exotropia [38]. Indeed, the authors suggested that reading and visual–motor integration deficits may be causally linked. Also, in children with intermittent exotropia, Fang et al. [39] linked poor stereo function at near to slower reading. However, others have found that reading ability is not reduced in children with intermittent exotropia [40].

Although most studies find that reading ability is affected in amblyopia and/or strabismus, the evidence that this translates into poorer academic performance is extremely limited. Horvat-Gitsels et al. found no association between a history of amblyopia and either adverse performance or age-related attainment trajectories in core subjects during key stages of statutory schooling [41]. Also, in a large-scale UK study in adults, amblyopia was found to have no impact on educational, employment-related or economic outcomes [42]. It is important to point out that even where reading ability is reduced in individuals with amblyopia and/or strabismus, this demonstrates association and not necessarily causation. In other words, any deficit in reading in an individual with amblyopia or strabismus should not be automatically attributed to the visual condition.

#### Postural control

It is becoming increasingly apparent that the development of motor ability is affected by the presence of amblyopia and strabismus. In Zipori et al. [43], balance was found to be affected when binocularity is compromised and Kelly et al. [44] observed a range of motor ability deficits in their large sample of children with strabismus and anisometropia. However, postural control was affected only in the children who were amblyopic; balance was not significantly different in the non-amblyopic children with strabismus relative to the controls [44, 45]. The finding that balance is reduced in amblyopia and strabismus was supported in a recent narrative review of the literature [46]. Although the presence of amblyopia seems to be an important determinant of whether balance deficits exist, some inconsistencies are present in the literature. For instance, Capar et al. found improvements in dynamic balance control in children with strabismus following therapeutic surgery [47].

#### Other motor control deficits

Alongside balance deficits, Kelly et al. described poorer performance in manual dexterity, aiming and catching tasks in children with amblyopia (strabismic and/or anisometropic) compared to control children [44]. However, in the children with these conditions who were non-amblyopic, performance was worse for the manual dexterity task only.

Webber et al. also found reduced fine motor skills in children with amblyopia, particularly in strabismic amblyopes and for manual dexterity tasks requiring speed and accuracy [48]. In another study of motor control in amblyopes, the magnitude of the deficits was found to be correlated with the loss of stereoacuity, but not with the severity of the amblyopia [49]. A similar conclusion was reached by Hou et al. [50]. Jost et al. found impaired motor skills in children with intermittent exotropia [40].

#### Associations with physical activity

Alongside the better-known impacts of amblyopia and strabismus, recent studies suggest that children with these conditions may differ from visually normal children in unexpected ways. For example, Lee et al. reported that amblyopic adolescents may present with lower energy expenditure and a higher body-mass index [51]. However, this is a contested finding. Koritala et al. found that despite significant impairments in motor skills and differences in athletic self-perception and functional vision, children with amblyopia maintain overall physical activity levels and comparable body-mass indices relative to their peers [52].

#### Psychosocial Impacts

While the psychosocial impacts of amblyopia, strabismus and the treatment of these conditions have been studied for a considerable time (e.g. [53, 54]), there are some inconsistencies in the literature and this is leading to continuing research and a number of interesting new developments. Webber et al. found that children treated for amblyopia had significantly lower social acceptance scores compared to age-matched control children, but other self-perception scores (e.g., relating to scholastic or athletic competence or physical appearance) were not reduced compared to the controls [53]. Within the amblyopic group, a lower social acceptance score was significantly correlated with a history of patching for amblyopia, but not with a history of strabismus or wearing glasses. Many other studies have found that strabismus is associated with significant, negative psychosocial consequences [55–58]. It is important to point out that whenever psychosocial impacts are demonstrated, it may be that these stem from the treatment for the condition (e.g., patching) rather than from the condition itself. Equally, it may be that the impact stems solely from an awareness by the individual that their eye condition makes them different from their peers in some way.

Birch et al. reported lower self-perception of peer acceptance and physical competence in children with amblyopia, and that the self-perception of physical competence was correlated with aiming and catching skills and stereoacuity [59]. This is interesting because whilst the psychosocial impact of amblyopia and strabismus (and the functional impact of these conditions upon visuomotor control) are both well established, evidence is now emerging to suggest that the psychosocial effect size and visuomotor deficits may be correlated with one another [35, 58]. Also, it appears that these deficits are not predictable from clinical measures of vision [35]. In a more recent study by the same authors [60], psychosocial vision-related quality of life scores were associated with the presence of strabismus. Jost et al. found, amongst other deficits, reduced quality of life and self-perception scores in children with intermittent exotropia [40].

Consistent with the evidence that the presence of strabismus has a marked psychosocial impact (e.g., [61, 62]), recent evidence from Ehlers et al. [63] indicates that psychosocial factors can have a significant impact on expectations of strabismus surgery and that surgery can lead to significant improvements in several psychosocial domains. For example, Alam et al. noted improvements in both psychosocial and functional domains following strabismus surgery [64].

#### Other effects

The results of Dias Rohr et al. indicate impairment in the perception of geometric illusions (e.g., the Ponzo illusion) by children with strabismus [65]. Recently, Rakshit et al. reported that, during binocular viewing, individuals with amblyopia and/or strabismus take longer to complete visuo-cognitive tasks (e.g., the Stroop test) compared with normally sighted controls, and that pronounced differences also exist in executive functions (e.g., cognitive flexibility) [60].

The range of tasks for which deficits are present amongst individuals with amblyopia and/or strabismus appears to be undergoing a rapid expansion. For example, in a random sample of US Medicare beneficiaries aged 65+ years with a disorder of binocular vision, a retrospective study found significantly higher odds of sustaining a musculoskeletal injury, fracture or fall [66].

Box 1 Summary of findings relating to the prevalence and impact of strabismus and amblyopia Prevalence

- The prevalence of strabismus and amblyopia continues to be studied intensively.
- Recent studies shed light on the prevalence of these disorders in different geographical regions, amongst different ethnicities, and in both clinical populations and unselected populations.

Impact

- The impact of living with amblyopia and strabismus includes the obvious impact upon clinical visual measures, but extends into fine motor control, including balance.
- While reading ability may also be affected, evidence for an impact on academic performance is weak or non-existent.
- Distinguishing between the impact of strabismus and amblyopia when they co-exist is challenging. Even in the absence of strabismus, the lived experiences of people with amblyopia indicate that the quality-of-life impacts of the condition are substantial and varied.
- There is growing evidence of a robust psychosocial impact of both amblyopia and strabismus, with strabismus seemingly having a more marked impact upon self-perception.

### New developments in investigation of strabismus and amblyopia

#### Smartphones and digital interventions

Digital interventions aimed at prevention or treatment are a rapidly expanding field [67]. Now approaching ubiquity in the developed world [68], smartphones can be used increasingly to provide intervention [69, 70] and their widespread availability may tackle some of the accessibility barriers in reaching underserved populations.

Ophthalmology has seen significant research interest in this area [71, 72], with many smartphone apps targeting amblyopia, which has largely been the main condition of interest [71]. The affinity that many young children may have with these devices may break down some of the barriers existing in examining young patients [72], and remote consultation may enable the collection of additional data outside the clinic for better risk stratification. A few areas explored in the literature are reported below. The reader is referred to extensive reviews of the topic for more in-depth summaries [71, 72].

##### VA tests

Examples include traditional visual acuity (VA) charts (e.g., Peak Acuity, Peek Vision, peekvision.org) and other apps more specific to amblyopia monitoring [71, 72]. The latter includes the Kay Amblyopia Tracker (Kay Pictures, kaypictures.co.uk), a VA measurement app using Kay Pictures, which provides parents with a method to monitor VA [73].

##### Stereoacuity test

The Stereoacuity app (University of Bergamo, unibg.it) requires anaglyph glasses and showed 95% limits of agreement of ~0.65 log_10_ arcsec with the TNO stereo test [74]. Other work by Yang et al. [75] featuring 3D technology (autostereoscopic monitor via parallax barrier technology [76]) showed good agreement with the Lang and Random dot tests (all kappa ≥ 0.84) for pass/fail criteria. The high resolution of modern smartphone screens represents an important advance in making this 3D technology more usable, owing to the fact that stereo-limits depend on pixel resolution [75].

##### Amblyopia

The “GoCheck Kids” app (Gobiquity, gocheckkids.com), to be used by paediatricians, aims to detect amblyopia and strabismus from 6 months through 6 years of age. This is a US Food and Drug Administration (FDA)-approved photo screening app that uses the phone’s built-in camera and flash to induce a red reflex, which is then interpreted by the software to determine the presence of refractive errors. Strabismus is assessed by comparing the position of the corneal reflexes. Also, VA measurements can be performed. The app provides an outcome based on the presence of amblyopic risk factors and has shown acceptable specificity and variable sensitivity [77–79]. The accuracy of determining refractive errors, particularly astigmatism, has been questioned, and may depend on the direction of the flash-camera displacement [72]. Overall, unsupervised digital amblyopia screening has gained interest, as this could be adopted at home and may overcome some of the barriers of the conventional healthcare practitioner-delivered screening, such as cost, accessibility and the limited number of qualified screeners [80]. A systematic review on this topic included 29 studies assessing home-based, unsupervised amblyopia screening [81]. The authors found a wide range of sensitivities and specificities across and within modalities. A meta-analysis was not conducted due to the high heterogeneity. Additionally, 52% of studies were found to have potential selection bias concerns from the recruitment methods employed. Despite genuine potential from existing devices and software, there is a strong need to improve the quality of diagnostic accuracy studies in this area to evaluate the suitability of proposed devices to achieve their stated aims [81, 82].

##### Strabismus assessment

The reference standard for strabismus angle assessment is the prism cover test, which requires extensive clinical experience for accurate measurements [83]. Access to this expertise may vary based on geographic location and socio-economic status [84, 85]. Accurate and automated quantification of strabismus would have the benefit of increasing accessibility to diagnosis, while also enabling more objective metrics in this field [86]. Additionally, assessment becomes more challenging in the case of uncooperative patients, often triggered by an unfamiliar clinical environment for young children. Smartphone apps used in the home setting may prove advantageous to enhance paediatric strabismus examination [87]. Several applications have been proposed for this purpose, including StrabisPIX (Boston Children’s Hospital, accelerator.childrenshospital.org) [88] and EyeStrab (EyeStrab, eyestrab.com) [89], which aim to assess strabismus from photographs. StrabisPIX guides patients to position their eyes in the nine cardinal directions of gaze, after which the images are interpreted by clinicians. The quality of patient-taken pictures was comparable to those taken in clinical settings, and the app may facilitate faster data collection in the clinic [88]. EyeStrab detects and calculates the angle of strabismus from photographs, and it was validated in a cohort of 72 patients with strabismus. The study showed high correlation with clinicians’ measurements, but large 95% limits of agreement of ±15 Δ [89]. Similar to the above, the EyeTurn app [90] is a photographic app that conducts an automated Hirschberg test by automatically detecting centration of the corneal light reflexes, compared to the pupil centres. Despite a strong correlation with the prism cover test in a group of strabismic patients (*R*^2^ = 0.95), no Bland–Altman agreement analysis was conducted [90]. There are additional reports of apps using the smartphone’s built-in camera to achieve objective assessment of strabismus via corneal reflex detection [91, 92]. Overall, this does not in itself represent a novel invention, but rather the automatisation of well-established techniques; therefore, it is important to recall known limitations of corneal reflex-based strabismus measurements, such as poor fixation and variability in angle kappa. Having these techniques available in smartphone devices offers some accessibility and cost advantages compared to dedicated devices [93], but agreement with the reference standard either remains poor or inadequately explored.

Despite the significant clinical potential of smartphone-based apps, several issues and concerns require further evaluation before wider clinical utilisation. Notably, only a minority of available studies have evaluated usability or participant satisfaction [71]. This requires thorough examination in representative populations before the wider use of such devices can be undertaken. Additionally, despite most existing applications showing potential clinical utility in ophthalmology, several devices exhibited *lower* diagnostic accuracy and disease detectability compared with conventional examinations, and thus further refinement may be necessary [71].

Beyond smartphone-based approaches, retinal birefringence scanning is a relatively new technology for assessing fixation accuracy and ocular misalignment, based on the birefringent properties of the Henle fibre layer surrounding the fovea [94]. Devices such as the Blinq (Rebion, rebion.co) are portable, non-invasive tools designed to screen for strabismus (including small-angle deviations) by determining whether a patient can consistently align both visual axes on a target. If not, the individual may require further investigation. A number of studies have evaluated the Blinq’s accuracy in amblyopia and strabismus screening, with mixed results [95–97].

##### Virtual reality (VR) visors

Several VR visors have been developed in the field of ophthalmology, with amblyopia assessment and management representing a key area of focus [98]. Examples are the Luminopia One (Luminopia, luminopia.com), an FDA-approved wearable VR headset for amblyopia treatment in children aged 4–12 years, which has shown significant VA improvement [99] and the CureSight (NovaSight, nova-sight.com), another VR headset proposed for amblyopia treatment. Beyond amblyopia management, these devices may allow strabismus measurement and they may help to establish whether intervention may be beneficial [100]. Alternative approaches include devices such as the Pivothead (Pivotvision, pivotvision.com). They feature glasses equipped with a camera, to be worn by clinicians, enabling hand-free recording of ocular motility and strabismus assessment. These could be valuable for documentation purposes, especially when measuring the strabismus angle while holding a target, occluder and prism bar [98]. Despite VR headsets being another rapidly expanding field, several constraints or grey areas require further consideration and development. A key consideration is the device cost, which may remain a major limitation for some of these tools [98]. Also, the devices reported in the literature are rarely validated in large and representative groups of patients [98]. Importantly, age-specific validation is required to ensure accuracy in the relevant age group, and often this is lacking [72]. Lastly, similar to smartphones, usability and user experience are seldom measured [71].

#### Artificial intelligence

Artificial intelligence (AI) may have a significant role in strabismus care, particularly in relation to screening and diagnosis [101]. AI may provide enhanced analyses of eye movement videos captured with eye tracking, resulting in a more patient-friendly assessment, thus potentially reducing some of the stress arising from conventional procedures. AI analysis of eye movement videos gathered with several devices, such as VR systems, eye trackers, and digital analysis of cover testing, demonstrated high diagnostic accuracy in experimental settings for several strabismus types [102–104]. Images of ocular position from photographs have also been considered, and this approach has greater potential than eye tracking, in that it is more accessible and more rapid, and specialist equipment is not required.

Reviews of this topic have generally grouped AI software under machine-learning and deep-learning headings [101, 105]. More traditional machine-learning approaches employed methods based on facial photographs and machine-learning algorithms that often included some preliminary steps of segmentation of ‘region of interests’ (facial and eye landmarks), such as the whole cornea, the corneal reflexes or the pupil centre, which are then used to quantify any eye deviation. Generally, these methods remain easier to interpret and are less computationally demanding. Several examples are reported in the literature, with varying levels of diagnostic performance (see Wu et al. for a review [101]). Newer approaches employing deep learning are becoming increasingly explored. These learn directly from raw data, without predetermined feature extraction, generally leading to higher diagnostic accuracy but limited interpretability [101]. For example, Zheng and colleagues developed a deep-learning algorithm using a primary gaze position photograph to detect horizontal strabismus, achieving a sensitivity and specificity of 0.94 and 0.99, respectively, in an external validation set, which was better than that of the resident ophthalmologists [106]. Notably, many types of more complicated strabismus/conditions were excluded (e.g., restrictive strabismus, sensory strabismus, paralytic strabismus, myasthenia gravis, nystagmus and Duane syndrome). These findings and limitations are similar to those described by Mao et al. [107].

A recent meta-analysis of 24 studies found AI models to exhibit strong diagnostic performance, with a pooled sensitivity and specificity of 0.94 and 0.94, respectively, for the presence of strabismus [105]. While performance appeared strong, over half of the studies had a ‘high’ or an ‘unclear’ risk of bias. Also, heterogeneity was high, and thus the generalisability of findings remains questionable. As noted above, limiting the types and range of strabismus included was common across studies, which is likely to lead to an over-estimation of accuracy. Current methods may perform well in establishing whether more basic types of strabismus are present or absent, while being less accurate in determining the type and characteristics of the deviation [105].

Several groups have reviewed the field of automated assessment of strabismus, including AI-based methods [86, 108]. Overall findings indicate wide-ranging sensitivities and specificities, along with high heterogeneity, reflecting significant differences across samples, methodology and the technology itself. However, high correlation and acceptable diagnostic accuracy for automated methods of strabismus detection make this a promising area of development that requires further investigation.

#### OCT

Optical coherence tomography (OCT) can provide an in-depth analysis of structural changes in the anterior visual pathway of amblyopic eyes, and could represent a tool for more thorough investigation of strabismus and fixation behaviour.

Several groups have evaluated OCT structural findings in amblyopia, particularly as changes in retinal layer thickness in the macula [109–111]. Results are conflicting and evidence showing greater thickness in the amblyopic compared with the fellow eye is poor [109–111]. Optical artefacts arising from differences in refractive error and the use of different OCT devices may explain differences between studies [109]. Importantly, some OCT results of a thickened retina contrast with histological findings from animal models of amblyopia, which reported reduced ganglion cell density after deprivation [112–114].

The choroid has also been studied, due to the emphasis on its role in emmetropisation and responsiveness to defocus [115, 116]. Meta-analyses are concordant in reporting increased choroidal thickness in the amblyopic eye compared with both the fellow and control eyes [109, 111]. However, prospective studies assessing changes in choroidal thickness after amblyopia treatment are conflicting, with reports of normalisation of thickness compared to the fellow eye in some studies [117, 118] but not in others [119, 120].

Multiple systematic reviews consistently demonstrate that amblyopic and fellow eyes exhibit reduced macular retinal vessel density compared to healthy controls, though it remains unclear if these microvascular changes are a cause or a consequence of the condition [121–123].

Fixation accuracy and stability have been assessed with the OCT [124–126]. A well-established characteristic of amblyopia is the inability to maintain accurate and steady fixation [127], which has been observed and characterised with multiple structural and functional modalities [128, 129]. Clinical assessment and quantification of fixation accuracy and stability require infrequently used subjective measurements such as Haidinger’s brushes, Visuoscopy [13] or microperimetry. Plaumann et al. estimated fixation accuracy and stability through serial OCT scans in a study of 30 adults with amblyopia and 30 controls [126]. The distance between the centre of scan and the fovea indicated fixation accuracy, while stability was estimated from the elliptical area of fixations over multiple acquisitions. Consistent with previous literature [124, 125], amblyopic eyes showed greater position errors compared to fellow and control eyes, as well as greater fixation instability. Both accuracy and stability were significantly related to VA [126]. Using OCT for this purpose may allow the magnitude of eccentric fixation in amblyopia to be estimated, with significant potential for examining whether it changes following treatment.

Beyond the retina, anterior segment OCT has been utilised to estimate extraocular muscle insertions during pre-operative assessment for strabismus surgery. While the modality shows good agreement with the reference standard of intraoperative callipers in first operations, evidence is insufficient in cases of re-operation, where the clinical utility would be most significant [130].

### New developments in treatment and disease understanding of strabismus and amblyopia

#### Strabismus

A 2017 overview of Cochrane reviews concerning interventions for binocular vision anomalies found that many of the 12 reviews available for strabismus remained inconclusive, with high uncertainty due to a high risk of bias of the available randomised controlled trials (RCTs). For strabismus, the best available evidence came from non-randomised studies, highlighting the clear need for more high-quality RCTs [131]. The availability of high-quality evidence to guide clinical practice in strabismus has only improved slightly over the last decade, as evidenced in the recent preferred practice pattern (PPP) guidelines for Esotropia and Exotropia issued by the American Academy of Ophthalmology (AAO) that still indicated many areas of treatment are guided by expert consensus and a lack of solid RCT-driven evidence to guide practice [1]. Similarly, more recent Cochrane reviews on strabismus management are unable to provide definitive conclusions due to paucity or low quality of the RCTs [132–134].

#### Intermittent exotropia

Guidelines from the AAO on intermittent exotropia provide a clear recommendation on the importance of monitoring children with this condition [1]. Indeed, recent work from the Paediatric Eye Disease Investigator Group (PEDIG), provided valuable insights into the natural history (over 3 years) of intermittent exotropia in children aged 3–10 years. In this group of untreated patients where surgery was not considered immediately necessary at baseline, deterioration of intermittent exotropia was uncommon and deviation control remained stable or slightly improved over the 3-year period [135]. The guidelines advise treatment in cases where fusion control remains suboptimal despite the correction of any refractive error [1].

A conservative treatment modality that has been increasingly evaluated is to use additional minus lenses to stimulate accommodative convergence [1], although the actual mechanism of action may, in fact, be more complicated. Indeed, some investigators have suggested that fusional convergence may be a main force at play in the control of intermittent exotropia, resulting in excessive accommodation. Accordingly, over-minus lenses may compensate for the excessive accommodation and restore clear vision, rather than stimulating accommodation in the first place [136]. This represents an area of active investigation and development, particularly supported by increased capability of continuous tracking of vergence and accommodative responses to improve assessment of oculomotor behaviour [137]. Work from another group suggested that proximal convergence may contribute significantly to exotropia control, with neither accommodative convergence alone nor fusional convergence being primarily responsible [138]. In this study, many patients with intermittent exotropia were capable of retaining eye alignment while also preserving clear vision [138]. Despite the fact that the exact mechanism of action remains unclear, over-minussing was reported as being the most effective treatment in improving near and distance control in a recent meta-analysis [139]. While RCT evidence suggests that over-minussing improves the control of intermittent exotropia [140–142], there remain two significant unanswered queries requiring clarification [139, 143]. These relate to the deterioration of gained control after discontinuation of treatment [142] and to the increased risk of inducing myopia progression [1, 144]. Overall, therefore while much has been done in exploring treatment modalities for intermittent exotropia, many questions remain unanswered [134, 139, 143].

##### Bupivacaine for strabismus treatment

Bupivacaine is a local anaesthetic which can alter the elastic and contractile properties of the extraocular muscles [145–147]. The treated muscle becomes hypertrophic, with sustained greater contractility in the muscle’s field of action and increased stiffness in the opposite field [148–151].

Bupivacaine can be utilised alone or in combination with botulinum toxin in the antagonist muscles [152]. A recent UK, retrospective study reviewed patients with age-related distance esotropia treated either with surgery or Bupivacaine [153], and showed similar success rates (63% and 64%). Bupivacaine may represent a viable option to manage small angle deviations (≤15 Δ), in patients not tolerating prisms or where surgery may be more risky or unsuitable [154, 155]. Despite being a promising option, larger prospective RCTs are needed to assess treatment dose and longer-term follow-up for efficacy and safety across different types of strabismus [153, 154].

#### Amblyopia

The overview of Cochrane reviews described above found better evidence to support amblyopia interventions compared with those suggested for strabismus [131]. This is reflected in the AAO’s guidelines for amblyopia treatment, whereby evidence informing treatment selection is often stronger based on the available RCTs and meta-analyses [156]. The last two decades have been instrumental in making amblyopia care more evidence-based, particularly with work from PEDIG, which has informed several changes in treatment paradigms and whose clinical trials have reduced the treatment burden of amblyopia by reducing the number of hours of patching and the frequency of administering atropine eye drops [157].

It is well established that early detection and prompt treatment are essential for amblyopia treatment success, that treatment should be offered to children regardless of age (including teenagers) and that treatment success decreases with age [156]. Guidelines state firmly how optical correction of significant refractive errors alone, over a period of continued wear of ≥18 weeks, is the initial step in amblyopia care [156]. Often this step alone enables significant visual improvement and normalisation of VA, with meta-analytic evidence indicating how significant VA improvements are achieved in anisometropic, strabismic and combined-mechanism amblyopia [158]. Given the results of glasses-only treatment, it has become a reasonable consensus to prescribe glasses initially and then follow the patient with this treatment until no further improvements are seen [157].

Conventional treatments, such as occlusion and atropine penalisation, have been shown to be equivalent to one another in improving VA in well-conducted RCTs [159]. However, the success of these conventional options is limited, with up to one in three children not achieving a VA of 0.20 logMAR or better in the amblyopic eye, and only limited improvement in stereopsis [160, 161]. Thus, alternative treatments for amblyopia may be valuable and have been explored extensively [162].

##### Dichoptic therapy

Dichoptic or binocular therapy for amblyopia is among the most explored alternative treatment approaches, with a growing body of evidence including RCTs. Dichoptic contrast balancing, first introduced by Hess et al. in 2010 [163], consists of the presentation of distinct visual stimuli to the two eyes. Stimuli are modulated binocularly to encourage the use of the amblyopic eye, while simultaneously reducing input to the dominant eye [164]. Treatment can be delivered in many ways while viewing videos or playing video games, and through devices such as polarising/anaglyph glasses or VR visors.

In a technology report and in the PPP guidelines, the AAO commented on how the promising findings in early non-randomised studies were not substantiated in subsequent RCTs [156, 165]. RCTs assessing second-generation binocular therapy also showed contrasting results, but software and hardware development have continued and have been linked with improved outcomes. A 2022 Cochrane systematic review on dichoptic treatments in children with amblyopia compared efficacy to conventional patching or pharmacological treatment. Notably, to mirror real clinical scenarios, the studies that were included were restricted to those that started the additional treatment after refractive adaptation, i.e., after the use of glasses alone for at least 8–16 weeks [166]. The search was conducted in 2020, with only one eligible study [167], limiting robust conclusions [166]. Since 2020, additional RCTs have evaluated dichoptic treatments and recently, at least four new systematic reviews and meta-analyses on the topic have been published, utilising less stringent inclusion criteria than the Cochrane review described above [168–172]. A summary of findings of those comparing dichoptic training to occlusion is reported in Table 1.

**Table 1:** Summary findings of recent systematic reviews and meta-analyses evaluating dichoptic therapy compared with standard occlusion in children with amblyopia [168, 169, 171].

Details of available studies	• Number of included RCTs in reviews ranged between 12 and 14. • Age of participants in RCTs ranged between 5 and 14 years. • Follow-up time in RCTs ranged from 2 to 24 weeks, with a mean of ~10 weeks. • Total dichoptic therapy duration in RCTs ranged from 6 to 168 h with a mean of ~58 hours.
Visual acuity outcomes	• No significant differences in VA improvements between treatments [168, 171], or patching found to be slightly better [169]; • Substantial heterogeneity in estimates (*I*^2^, range: 44–85%), with contributing factors being treatment modalities/protocols, amblyopia subtypes, baseline severity, patient age and prior treatments. • Subgroup analysis of VA outcomes by high adherence to dichoptic therapy (>60%, *n* = 9/14 studies) showed a significant benefit compared with patching in one meta-analysis (SMD: −0.59, 95% CI −0.98 to −0.20; *P* < 0.01; *I*^2^ = 73%; very low certainty) [171]. • More hours of dichoptic therapy were significantly associated with lower improvement [171]; this apparently counterintuitive result may be partly explained by a ceiling effect with dichoptic therapy.
Stereoacuity	• Consistently across reviews, there were no significant differences in stereopsis gain between dichoptic therapy and patching.
Adverse events	• Adverse events and complications (e.g., new incidence/worsening of heterotropia and diplopia) were reported by a minority of included RCTs but were found overall to be similar between treatments [168, 171].
Risk of bias	• Consistently across reviews, the risk of bias was unclear for many included studies and most had some concerns. There were concerns about the available quality, leading to low certainty clinical recommendations.

*RCT* randomised controlled trial, *SMD* standardised mean difference, *V*A visual acuity

Recent systematic reviews concluded consistently that current dichoptic therapies may have similar or slightly lower efficacy compared to conventional therapies for VA improvement. There is also consistency in reporting similar improvement in stereopsis. However, the certainty of the evidence remains low due to the risk of bias of the included studies and because of high heterogeneity. As expected, also for dichoptic therapy, adherence seems to be a key element and obtaining high adherence and engagement appears critical for any device to enable treatment success [166, 168–171]. This is not without challenges, especially when engagement for dichoptic amblyopia treatments competes with currently available and always evolving video game units and content [165].

If dichoptic therapy is to become a viable alternative to the standard treatment of amblyopia, several knowledge gaps need to be closed. Areas of consideration include better stratification of results by age and amblyopia subtype [168, 170], more standardised treatment regimens [156, 168, 170] and consistent and objective reporting of adherence [167, 171, 173]. Efficacy across different strabismus types and magnitudes should also be clarified, as current delivery devices may be better suited to small-angle deviations and may not present stimuli accurately in cases of larger deviations. Costs and availability may be limiting factors of these technologies, especially when compared with the proven efficacy and affordability of patching and atropine eyedrops [165]. Cost-effectiveness considerations are essential, considering not only equipment costs but also the significant time commitment. Use in outpatient settings adds travel time and expenditure, increasing patient burden and reducing acceptability [174, 175], while even home-based use typically requires at least an hour per day, potentially diverting children from academic and other activities.

##### Non-invasive brain stimulation

Electromagnetic stimulation (EMS) has been explored in adult patients with amblyopia to enhance neural plasticity and produce visual improvement beyond the limits of the plastic period [176]. EMS aims to induce molecular responses to increase neural plasticity via the delivery of weak electric or magnetic pulses to determined cortical regions [177]. However, the exact mechanism of action remains unclear [164].

A recent systematic review evaluating EMS in adults found improvements in VA, contrast sensitivity and stereopsis, but highlighted how current studies are limited by small sample sizes and short follow-up durations [164]. The efficacy of EMS interventions needs to be validated in larger, prospective RCTs that include clearer treatment protocols, proposed mechanisms of action and long-term effects on visual function [164, 176].

The potential risks of non-invasive EMS in visual rehabilitation are low but also need to be considered carefully, particularly because these devices are utilised increasingly in the home setting [176, 178, 179].

##### Selective serotonin reuptake inhibitors

Pharmacological approaches to enhance plasticity in the adult brain for amblyopia treatment are being explored, with selective serotonin reuptake inhibitors representing a relevant example [180, 181]. A systematic review of four RCTs using fluoxetine or citalopram found a statistically significant improvement in VA of only 0.09 logMAR, which is of questionable clinical significance [182].

##### Perceptual learning for amblyopia

Perceptual learning has also been proposed for amblyopia treatment, beyond the plastic period [183]. The technique involves repetitive practice on targeted visual tasks to enhance specific visual functions, often resulting in long and intensive sessions [184, 185].

Two main types of perceptual learning have been proposed for amblyopia treatment [183], under monocular (amblyopic eye) and dichoptic conditions. Rigorously conducted trials in perceptual learning are needed as currently, many studies lack a control group or the application of a method of masking to reduce the risk of bias. Also, the issue of whether treatment outcomes are sustained in the long-term is often overlooked [156, 183].

##### Outcome measures for amblyopia

VA remains the only outcome measure reported in the majority of amblyopia studies; stereopsis, adverse events and health-related quality of life, despite being recognised as key outcomes, are much less frequently reported [186].

Recent laboratory studies have emphasised how amblyopia is primarily a loss of binocular vision [187]. Accordingly, it is argued (e.g., by Hess [162]) that clinicians should aim to treat the primary disorder (binocular suppression), and this is becoming possible with more recent binocular treatments. Importantly, when measuring outcomes, since monocular VA does not reflect the main source of the disorder found in amblyopia, binocular endpoints may be more suitable measures [187, 188]. However, current methods may be inadequate for the appropriate assessment of binocular vision, and it is likely that new clinical tests will be necessary [162]. In fact, stereopsis is the only test used routinely to quantify binocularity and while available tests can establish the presence of stereopsis, they may be less suitable for determining its change quantitatively over time, e.g., as treatment progresses [162, 189]. Newer stereoacuity tests are required, capable of measuring disparity outside the deemed normal range [162], to open the possibility of more accurate monitoring of changes in stereopsis over time (e.g. [190]). Tests for assessing lower-level binocular functions (e.g., contrast combination [191] or motion coherence [192]) may also be valuable in amblyopia treatment to provide much-needed low-level assessment of binocular function and monitoring of recovery.

## Non-strabismic binocular vision anomalies

### Prevalence of NSBVA

While the prevalence of amblyopia and strabismus continues to be the subject of considerable research interest, comparatively speaking, the prevalence of other binocular vision anomalies has received much less attention. Of the studies of these conditions that have been conducted, the results reveal a surprisingly high prevalence. However, results need to be interpreted with caution because often a single prevalence figure is quoted to indicate the combined prevalence of the various binocular conditions that come under the heading of ‘non-strabismic binocular vision anomaly’ (NSBVA). Also, the broad term NSBVA is frequently used in the literature for vision disorders, which include both binocular (e.g., convergence insufficiency) and accommodative (e.g., accommodative insufficiency) abnormalities. Here, the term NSBVA is used for binocular conditions which are non-strabismic, non-amblyopic and do not include disorders of accommodation.

A 2025 systematic review of the prevalence of NSBVA and age-related changes in binocular vision among middle-aged and older adults found only eight studies that met the inclusion criteria, and only four of these eight studies reported the prevalence of specific conditions [193]. In addition, only two reported the results for the overall prevalence of binocular vision abnormalities [194, 195]. Specifically, in a small sample of only 73 patients with age-related cataract, Tan et al. [194] reported that 32.9% had NSBVAs. Leat et al. [195] noted overall NSBVA prevalences of 27%, 30% and 38% for individuals aged 60–69, 70–79 and over 80 years, respectively. Lai et al.’s [193] review concluded that NSBVAs are common in older adults and that they become increasingly common with age. However, one of their main conclusions was that information about the NSBVA prevalence in this population remains very limited. Also, there are several limitations of the studies that have been published, including sample size issues and the diagnostic criteria employed.

In their 2011 review of both non-strabismic and accommodative disorders across all age groups, Cacho-Martinez et al. [196] identified only 10 studies that met their inclusion criteria. They indicated a lack of ‘proper’ epidemiological studies. The main issues highlighted included the population studied (selected versus unselected) and the lack of uniformity in diagnostic criteria, which contributed to wide-ranging and therefore highly uncertain estimates regarding prevalence. For example, the prevalence of convergence insufficiency was quoted as falling between 2.3% and 33%.

More recent studies continue to report on the prevalence of disorders in clinical populations [197–200] or amongst other selected populations [201–203]. Not surprisingly, such studies have tended to find extremely high prevalences of NSBVA.

In recent studies of unselected samples, NSBVA prevalence has generally been found to be lower. For example, Sánchez-Cuadrado et al. [204] reported a prevalence of convergence insufficiency of 5.6% in a sample of over 600 Spanish children aged 6–14 years and in an unselected sample of 175 university students. García-Muñoz et al. [205] reported an overall prevalence of NSBVA of 8%, with convergence insufficiency and convergence excess being the two most prevalent binocular conditions (3.4% and 2.3%, respectively). However, by contrast, in a sample of around 920 children aged 7–17 years in Tamil Nadu, Hussaindeen et al. [206] observed a prevalence of NSBVA around 30%, of which convergence insufficiency was the most prevalent (~17%). In that study, NSBVA prevalence was higher in the 13–17-year-old age group (36.2%) compared with 7–12-year-old children (25.1%). It is not clear why the Hussaindeen et al. estimate differs so much from the other reports from unselected samples, but the lack of standardised and diagnostically validated protocols for the assessment of NSBVAs is highly likely to have played a major role [207, 208]. Saikia et al. concluded that standardisation of assessment protocols and cutoff criteria is needed urgently to determine the prevalence of NSBVAs [207].

### Significance and impact of NSBVA

In contrast to the many studies that have assessed the impact of amblyopia and/or strabismus in various domains (e.g., functional, psychosocial), the impact of most NSBVA remains under-researched. The exception is convergence insufficiency, the most prevalent NSBVA. Goering et al. listed the following as commonly associated symptoms of convergence insufficiency [209, 210]: asthenopia, diplopia, headaches, blurred vision, movement of print while reading, and difficulty with reading comprehension, sleepiness and/or the inability to concentrate after short periods of reading or prolonged near work.

Since the symptoms arise at near, this is where the impact of the condition is most likely to be seen [211, 212], and the impact on reading performance is of particular interest [213]. Whereas some studies (e.g. [214, 215]) have not found a link between the presence of convergence insufficiency and reading performance, a number of investigations have reported such an association [201, 216]. This must be considered against weak evidence to suggest that reading ability is improved in individuals following treatment [215, 217, 218].

There are welcome developments in attempting to understand the mechanism by which convergence insufficiency, or indeed any NSBVA, may affect reading. For example, Gaertner et al. suggested that a close relationship between the saccadic and vergence systems for the binocular coordination of saccades may represent the underlying mechanism; the assumption being that the vergence disorder interferes with the saccadic eye movements needed for reading [219].

While there is broad acceptance that reading/academic performance may be reduced in convergence insufficiency, the nature of the relationship between the existence of the condition and reduced reading/academic performance is far from established [220], particularly in relation to whether the relationship is causative or associative. A wide range of issues have been identified, which makes it extremely challenging to understand the nature of the relationship. Issues include the fact that clinically observed convergence insufficiency is frequently asymptomatic and that a strong association exists between the presence of attention deficit hyperactivity disorder and convergence insufficiency, suggesting the possibility that both reading difficulties and convergence insufficiency may arise from an underlying neurological disorder [220]. Another issue relates to the non-specific nature of the symptoms of convergence insufficiency and, from the opposite perspective, the fact that the same symptoms may have other (i.e., non-convergence insufficiency) explanations. There is also an ongoing debate in the clinical literature about how convergence insufficiency is diagnosed (e.g., the number and nature of the signs needed to make a diagnosis [193, 211]), and about how symptoms should be established and their severity graded [221].

The Convergence Insufficiency Symptom Score (CISS) questionnaire has been developed to aid with convergence insufficiency diagnosis and to understand symptom severity [222]. However, a number of concerns have been raised about this instrument [223], specifically in relation to the predictive value of the symptom score for convergence insufficiency [224, 225]. Also, there appears to be no link between the clinical signs of convergence insufficiency and the severity of symptom scores obtained using this instrument [226]. Further, understanding the impact of convergence insufficiency is challenging because many non-visual factors can also influence reading and performance at school.

Box 2 Summary of findings relating to the prevalence and impact of non-strabismic binocular vision disorders (NSBVA) *Prevalence*

- The prevalence of NSBVA has received much less attention, and many studies suffer from major methodological limitations that greatly limit their usefulness. This leaves uncertainties regarding the prevalence of NSBVA.
- Even for the best-known NSBVA, i.e., convergence insufficiency, prevalence estimates continue to vary widely.

*Impact*

- The impact of NSBVA is less well understood because the symptoms are varied and not condition-specific.

### New developments in treatment and disease understanding of NSBVA

Compared with strabismus and amblyopia, there have been far fewer recent developments in the treatment and understanding of NSBVA. Reviews have shown how the signs and symptoms utilised as diagnostic criteria for vergence disorders (i.e., NSBVA) are variable and inconsistent, and mostly not supported by sound evidence [196, 208]. A systematic review from 2025 confirmed these findings, with highly variable and inconsistent investigation routines and criteria being used in the diagnosis of NSBVA [207].

Similarly, previous research has highlighted the limitations of the investigative techniques available, suffering from significant learning effects, variability, motivation (i.e., “try harder”), and lack of suitable normative data [227–231]. For example, a systematic review of normative data for fusional vergence [232] noted high heterogeneity in step vergence related to age, with significantly higher variability in children compared with adults.

#### Treatment

A few groups have explored the feasibility of delivering convergence training via VR visors in adults with convergence insufficiency and shown comparable improvements in motor outcomes compared to traditional, clinician-led programmes [233, 234].

#### Neural mechanisms

A number of studies, utilising MRI, are aimed at understanding the underlying neural basis for symptomatic convergence insufficiency and the effect of treatment [235–237]. However, research conducted so far does not allow any firm conclusions to be drawn as yet [238].

## Persistent issues and future directions

### Strabismus

Early detection and management of strabismus and other amblyogenic factors remains the best way to optimise the long-term sensorimotor outcome [1]. However, the quality of evidence informing strabismus management remains weak, with guidelines based on consensus rather than RCT evidence.

Two questions remain open in relation to the management of esotropia. The first is that while generally supported by expert consensus, the evidence supporting the efficacy of hyperopic correction in children to prevent the onset of strabismus remains poor [132]. More high-quality clinical trials are required to provide a better foundation for determining the ability of a spectacle correction to prevent strabismus in children with hyperopia. The lack of solid evidence concerning the effect of refractive correction is mirrored in current clinical practice guidelines relating to the prescription of hyperopic refractive errors, which show considerable variability regarding when to prescribe spectacles [132, 239].

The second question relates to the ideal timing of surgery for infantile esotropia, which was the subject of a recent Cochrane systematic review [133]. While early surgery has the advantage of better potential for binocularity and reduced muscle contracture, its planning faces more difficulty in obtaining reliable and accurate strabismus measurements. Late surgery has the advantage of making amblyopia management easier and can be based on more reliable measurements. This is counterbalanced by a reduced potential for binocularity, and the muscle contractures induced by strabismus can introduce a mechanical component to the deviation [133]. The topic is being studied [240, 241], but the literature on the timing of surgical interventions for infantile esotropia remains conflicting and continues to be hampered by the lack of RCTs.

Just as for esotropia management, appropriate optical correction of any significant ametropia is generally the accepted starting point in the management of intermittent exotropia, but there are no RCTs available detailing the efficacy of this treatment modality [143]. Many other questions remain to be answered regarding alternative treatment modalities for intermittent exotropia, such as the deterioration of gained control after discontinuation of over-minussing and the increased risk of inducing myopia progression [142, 144].

### Amblyopia

While the management of anisometropic, strabismic and mixed amblyopia has been investigated extensively [131, 159], there is a lack of evidence for the management of stimulus deprivation amblyopia [242]. Accordingly, realistic treatment outcomes, dose–response effects and precise treatment modalities are not yet clearly established.

With the newer approaches to amblyopia treatment (see above), it remains to be seen whether they deliver treatment outcomes that are faster or better (e.g., improved binocular function) than traditional amblyopia therapies; in particular, optical correction followed by patching [165].

### Non-strabismic binocular vision anomalies

Commenting on the 2020 Cochrane systematic review of therapy efficacy for convergence insufficiency [243], Rowe et al. highlighted how questions remain about which specific treatment procedures lead to better outcomes [244]. This is consistent with the 2021 AAO [245] report, which raised questions about the efficacy of office-based therapy for convergence insufficiency treatment and also regarding the effectiveness of home-based therapies. There are also questions about whether the treatments are as effective when delivered using low-tech, traditional target-based exercises, or whether there is an advantage to delivery on digital platforms [244]. Many RCTs have shown significant improvement in oculomotor outcomes, such as positive fusional vergence measures [246, 247]. This is not surprising, since the effect of motivation and effort on performance in this area is considerable [228, 230, 231]. However, improvement in symptoms has not been demonstrated consistently in adults [248, 249], and results remain mixed in children [247, 250]. Future studies are needed to determine which specific vergence and accommodative exercises are most efficacious. The optimal treatment dose (number of minutes per week) and duration (weeks of therapy) also require further investigation. A longer follow-up is necessary to determine if the treatment effects are maintained for more than 1 year. Aside from convergence insufficiency, no other NSBVA has been the subject of well-conducted RCTs [251].

In addition to issues relating to the treatment of NSBVA, there are several other basic points to address. These include the lack of normative binocular vision data in sections of the population [194, 227], which in turn connects to limited evidence on the prevalence of NSBVA, e.g., in middle- and older-aged populations [193]. There are also questions around the precise diagnostic criteria to use for each NSBVA [207, 208]. These are open to scrutiny because of the lack of clear links between the apparent presence of an NSBVA and symptoms and/or its impact (See above).

An additional issue is that the measures used to establish the effectiveness of vision therapy have yet to be established. A group of clinical procedures is used to identify and diagnose patients with NSBVA, and it is usual for these same measures to be used to assess treatment success. This risks introducing bias. The ideal measures for evaluating the success of treatment should be reliable and validated objectively; equally importantly, they need to be relevant to the patient [252]. There are a few psychometrically validated questionnaires for assessing symptoms [221], but generally they show poor specificity [224, 225] and may be more useful for monitoring change rather than helping with diagnosis [211].

## Final remarks and conclusions

For both strabismus and amblyopia, appropriate optical correction of any significant ametropia is the accepted starting point in their management [1, 156]. Despite recent improvements in the quality and availability of RCTs in the field of strabismus, several uncertainties and knowledge gaps persist in this area, with many clinical decisions informed by clinical consensus rather than RCT evidence. The quality of the evidence guiding amblyopia treatment is much stronger than that for strabismus. The prevalence of these conditions continues to command much research attention, but there has been a huge upsurge in the number of studies investigating the impact of living with them. The impact goes far beyond visual function and stretches into visuomotor task performance and notably into broader domains, e.g., psychosocial aspects.

NSBVA has received much less attention than amblyopia and strabismus, and while this is understandable because they are less serious conditions, their prevalence, impact and optimum treatment approach remain subject to considerable uncertainty.

Digital technologies offer considerable promise in the area of ophthalmology, including in binocular anomalies, particularly in relation to detection and treatment. However, it is by no means certain that they will transform the landscape of detecting and treating binocular disorders. To enhance adoption, the usability or participant satisfaction using the device or software is critical, and this is not a feature of many studies at present. Also, in relation to detection, a number of devices have exhibited *lower* diagnostic accuracy and disease detectability compared with conventional examinations [71].

We are currently in an unfolding story about how best to understand and treat binocular vision anomalies. With greater awareness of the need for robust evidence, the knowledge gaps are becoming clearer. The hope is that in the years ahead, the field of binocular vision research will continue to advance, further reducing the reliance upon clinical consensus for the management of these conditions.

## Data Availability

No datasets were generated or analysed during the current study.
